# 3-*O*-Methylfunicone, a Selective Inhibitor of Mammalian Y-Family DNA Polymerases from an Australian Sea Salt Fungal Strain

**DOI:** 10.3390/md7040624

**Published:** 2009-11-23

**Authors:** Yoshiyuki Mizushina, Hirohisa Motoshima, Yasuhiro Yamaguchi, Toshifumi Takeuchi, Ken Hirano, Fumio Sugawara, Hiromi Yoshida

**Affiliations:** 1 Laboratory of Food & Nutritional Sciences, Department of Nutritional Science, Kobe-Gakuin University, Nishi-ku, Kobe, Hyogo 651-2180, Japan; E-Mail: yoshida@nutr.kobegakuin.ac.jp (H.Y.); 2 Cooperative Research Center of Life Sciences, Kobe-Gakuin University, Chuo-ku, Kobe, Hyogo 650-8586, Japan; 3 Department of Applied Biological Science, Tokyo University of Science, 2641 Yamazaki, Noda, Chiba 278-8510, Japan; E-Mails: hirohisamtsm@yahoo.co.jp (H.M.); y.yamaguchi0623@gmail.com (Y.Y.); ttakeuch@rs.noda.tus.ac.jp (T.T.); sugawara@rs.noda.tus.ac.jp (F.S.); 4 Nano-bioanalysis Team, Health Technology Research Center, Takamatsu, Kagawa 761-0395 Japan; E-Mail: hirano-ken@aist.go.jp (K.H.)

**Keywords:** 3-*O*-methylfunicone, Y-family DNA polymerase, DNA polymerase κ, enzyme inhibitor, marine fungal strains, Australian sea salt, anti-cancer drug

## Abstract

We isolated a pol inhibitor from the cultured mycelia extract of a fungal strain isolated from natural salt from a sea salt pan in Australia, which was identified as 3-*O*-methylfunicone by spectroscopic analyses. This compound selectively inhibited the activities of mammalian Y-family DNA polymerases (pols) (*i.e.*, pols η, ι and κ). Among these pols, human pol κ activity was most strongly inhibited, with an IC_50_ value of 12.5 μM. On the other hand, the compound barely influenced the activities of the other families of mammalian pols, such as A-family (*i.e.*, pol γ), B-family (*i.e.*, pols α, δ and ɛ) or X-family (*i.e.*, pols β, λ and terminal deoxynucleotidyl transferase), and showed no effect on the activities of fish pol δ, plant pols, prokaryotic pols and other DNA metabolic enzymes, such as calf primase of pol α, human immunodeficiency virus type-1 (HIV-1) reverse transcriptase, human telomerase, T7 RNA polymerase, mouse IMP dehydrogenase (type II), human topoisomerases I and II, T4 polynucleotide kinase or bovine deoxyribonuclease I. This compound also suppressed the growth of two cultured human cancer cell lines, HCT116 (colon carcinoma cells) and HeLa (cervix carcinoma cells), and UV-treated HeLa cells exhibited lower clonogenic survival in the presence of inhibitor.

## 1. Introduction

We have long been interested in the integrity of the genome of eukaryotes and its relation to cell differentiation. DNA replication, recombination and repair in eukaryotes are key systems to maintain these processes [1], and DNA polymerases (pols) have important roles in these. In this regard, we have concentrated our efforts on investigating eukaryotic pols associated with these processes [2].

The human genome encodes at least 15 pols to carry out cellular DNA synthesis [3,4]. Eukaryotic cells contain three replicative pols (α, δ and ɛ), mitochondrial pol γ, and at least eleven non-replicative pols [β, ζ, η, θ, ι, κ, λ, μ, ν, terminal deoxynucleotidyl transferase (TdT) and REV1] [3–5]. Pols have a highly conserved structure, which means that their overall catalytic subunits vary, on the whole, very little among species. Conserved structures usually indicate important, irreplaceable functions of the cell, the maintenance of which provides evolutionary advantages. Based on sequence homology, eukaryotic pols can be divided into four main families, A, B, X, and Y [6]. Family A includes mitochondrial pol γ, and pols θ and ν, and family B includes three replicative pols (α, δ, and ɛ) and pol ζ. Family X is pols β, λ, μ, and terminal deoxynucleotidyl transferase (TdT), and family Y includes pols η, ι, κ, and REV1. Because not all functions of eukaryotic pols have been fully elucidated, selective inhibitors of pol families are useful tools for distinguishing pols and clarifying their biological functions. We have therefore been searching for natural compounds that selectively inhibit each of these eukaryotic pols [7–15], and we succeeded in identifying novel compounds with pol inhibitory activity from marine microbiological products [16–19].

In this study, we isolated fungal strains from Australian natural sea salt, and obtained 19 strains. These marine strains were screened, and a fungal strain displayed inhibitory activity against mammalian Y-family pols. The active compound purified from the strain, was identified as 3-*O*-methylfunicone, and the potential of this compound as an anti-cancer chemotherapy agent is discussed.

## 2. Results and Discussion

### 2.1. Purification of a DNA Polymerase Inhibitor, 3-O-Methylfunicone (Compound **1**), from a Marine Fungal Strain

**Figure 1.**
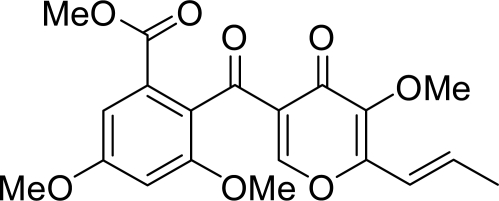
Structure of compound **1** (3-*O*-methylfunicone).

The 19 isolated and purified fungal strains from Australian sea salt were cultured, and each cultured mycelium was extracted with chloroform/methanol (v/v 1:1). The extracts were screened for inhibitory activity against mammalian pols, and the pol inhibitor (compound **1**) was purified by silica gel column chromatography from the active mycelium extract of “AF1-2 strain”. From the ^1^H- and ^13^C-NMR spectral data, compound **1** was identified as 3-*O*-methylfunicone (Figure 1).

### 2.2. Effect of Isolated Compound **1** on the Activities of DNA Polymerases and Other DNA Metabolic Enzymes

**Figure 2.**
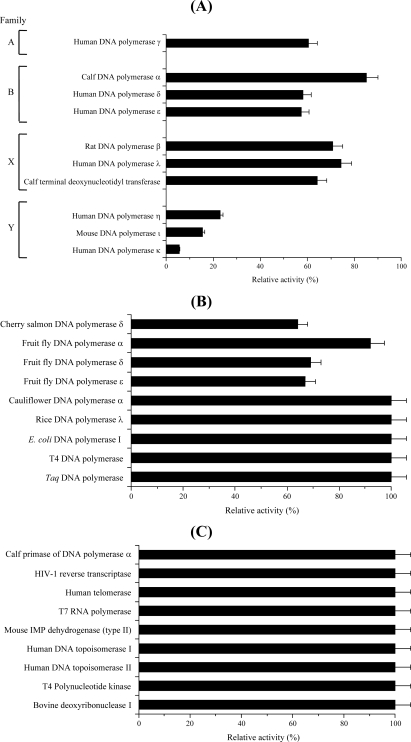
Effect of 3-*O*-methylfunicone (**1**) on the activities of various DNA polymerases and other DNA metabolic enzymes. (A) Mammalian pols, (B) fish, insect, plant and prokaryotic pols, and (C) other DNA metabolic enzymes. Compound **1** (100 μM) was incubated with each enzyme (0.05 units). % of relative activity. Enzymatic activities were measured as described in the Experimental section. Activities in the absence of the compounds were taken as 100%. Data are shown as the means ± SEM of four independent experiments.

**Figure 3.**
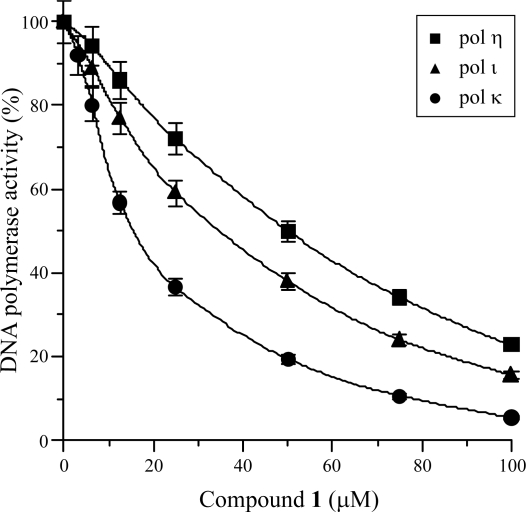
Mammalian Y-family DNA polymerase inhibition dose-response curves of 3-*O*-methylfunicone (**1**). Compound **1** was incubated with human pol η (square), mouse pol ι (triangle), and human pol κ (circle) (0.05 units of each). Pol activity was measured as described in the Experimental section. Activity in the absence of the compounds was taken as 100%. Data are shown as the means ± SEM of three independent experiments.

First, the isolated compound **1** was investigated as to whether it inhibited the activities of ten mammalian pols, such as families A (pol γ), B (pols α, δ and ɛ), X (pols β and λ and TdT), and Y (pols η, ι and κ). As shown in Figure 2A, this compound at 100 μM was found to selectively inhibit the activity of the Y-family of pols, and within this family pol κ was the most sensitive. Compound **1** inhibited the activities of these pols dose-dependently, and 50% inhibition of pols η, ι and κ was observed at concentrations of 50.1, 34.3 and 12.5 μM, respectively (Figure 3); therefore, the inhibitory effect on mammalian Y-family pols ranked as follows: pol κ > pol ι > pol η. On the other hand, the other families (*i.e.*, families A, B and X) of mammalian pols, fish pol (cherry salmon pol δ), insect pols (fruit fly pols α, δ and ɛ) hardly influenced the activities (Figure 2A and B), and higher plant pols (cauliflower pol α and rice pol λ), and prokaryotic pols (the Klenow fragment of *E. coli* pol I, T4 pol and *Taq* pol) had no effect (Figure 2B). When activated DNA (*i.e.*, DNA digested by bovine deoxyribonuclease I [DNase I]) and dNTP was used as the DNA template-primer and nucleotide substrate instead of synthesized DNA [*i.e.*, poly(dA)/oligo(dT)_18_] and dTTP, respectively, the inhibitory effects of the compound were unchanged (data not shown).

The same concentration (*i.e.*, 100 μM) of this compound did not influence the activities of other DNA metabolic enzymes, such as calf primase pol α, human immunodeficiency virus type-1 (HIV-1) reverse transcriptase, human telomerase, T7 RNA polymerase, mouse inosine 5′-monophosphate (IMP) dehydrogenase (type II), human topoisomerases I and II, T4 polynucleotide kinase, and bovine DNase I (Figure 2C). These results suggest that compound **1** may be a selective inhibitor of mammalian Y-family pols *in vitro*.

### 2.3. Mode of DNA Pol κ Inhibition by Compound **1**

**Figure 4.**
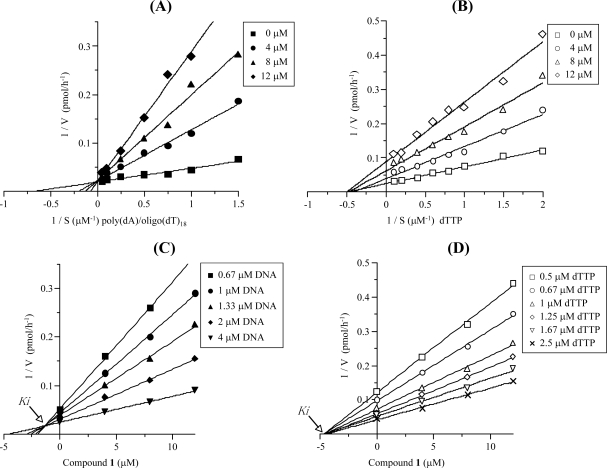
Kinetic analysis of the inhibition of human pol κ by compound **1**. (A) Human pol κ activity was measured in the absence (closed square) or presence of 4 μM (closed circle), 8 μM (closed triangle) or 12 μM (closed diamond) compound **1** using the indicated concentrations of the DNA template-primer. (B) Human pol κ activity was assayed with the indicated concentrations of the substrate dTTP in the presence of 4 μM (open circle), 8 μM (open triangle) or 12 μM (open diamond) or in the absence (open square) of compound **1**. (C) and (D) The inhibition constants (K_i_) were determined as 1.3 and 4.4 μM from a Dixon plot made on the basis of the same data for (A) and (B), respectively. Amount of human pol κ in the assay mixture was 0.05 units.

Next, to elucidate the mechanism of inhibition, the extent of inhibition as a function of DNA template-primer or dNTP substrate concentrations was studied (Figure 4). In kinetic analysis, poly(dA)/oligo(dT)_18_ and dTTP were used as the DNA template-primer and dNTP substrate, respectively. Double reciprocal plots of the results showed that compound **1** induced inhibition of human pol κ activity was competitive with the DNA template-primer and non-competitive with the dNTP substrate (Figure 4A and B). In the case of the DNA template-primer, the apparent maximum velocity (V_max_) was unchanged at 52.6 pmol/h, whereas the 295, 467, and 812% increases in Michaelis constant (K_m_) were observed in the presence of 4, 8, and 12 μM of compound **1**, respectively (Figure 4A). The K_m_ for the dNTP substrate was 2.00 μM, and the V_max_ for the dNTP substrate decreased from 41.7 to 11.1 pmol/h in the presence of 12 μM compound **1** (Figure 4B). The inhibition constant (K_i_) value, obtained from Dixon plots, was found to be 1.3 and 4.4 μM for the DNA template-primer and dTTP substrate, respectively (Figure 4C and D). When activated DNA and four dNTP substrates were used as the DNA template-primer and dNTP substrate, respectively, the inhibition of human pol κ by compound **1** was competitive with the DNA template-primer and non-competitive with the dNTP substrate (data not shown). These results suggest that compound **1** directly binds to the DNA template-primer-binding site of pol κ, whereas this compound may bind or interact with a site that is distinct from the dNTP substrate-binding site.

On the other hand, the inhibition of family B pols, such as pols α, δ and ɛ, by aphidicolin, which is a known classic pol inhibitor, was uncompetitive with activated DNA as a DNA template-primer and competitive with respect to the dNTP substrate [20]. Moreover, aphidicolin inhibited pol α by competing with dCTP but not by competing with the other three deoxynucleoside triphosphates [20]. In contrast, inhibition of pol κ by compound **1** was non-competitive with the four deoxynucleoside triphosphates (data not shown). The mode of the inhibitory effect of compound **1** on Y-family pols was quite different from the mode of the inhibitory effect of aphidicolin on B-family pols.

### 2.4. Inhibitory Effect of Isolated Compound **1** on Cultured Human Cancer Cells

Pols have recently emerged as important cellular targets for chemical intervention in the development of anti-cancer agents. The isolated compound **1** could therefore be useful in chemotherapy, and we investigated the growth inhibitory effect of this compound against two human cancer cell lines, a colon carcinoma cell line, HCT116, and a cervix carcinoma cell line, HeLa.

**Figure 5.**
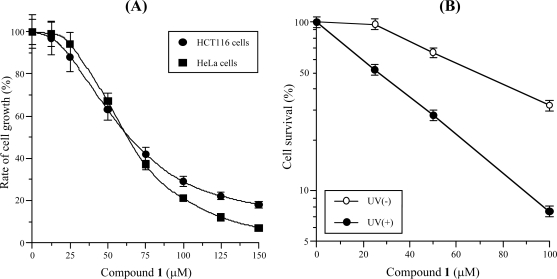
(A) Human cancer cell growth inhibition dose-response curves of 3-*O*-methyl-funicone (**1**). Various concentrations (0 to 150 μM) of compound **1** were incubated with human colon carcinoma (HCT116) cells (circle) and human cervix carcinoma (HeLa) cells (square) for 24 h. Rate of cell growth was determined by MTT assay [44] as described in the Experimental section. Data are shown as the means ± SEM of five independent experiments. (B) Clonogenic survival of UV-treated HeLa cells by compound **1**. Various concentrations (0 to 100 μM) of compound **1** were incubated with 10 J/m^2^ UV-treated (closed circle) or untreated (open circle) HeLa cells. After 7 days, the number of colonies in each treatment group was counted. Values are the means ± SEM of two independent experiments.

As shown in Figure 5A, compound **1** dose-dependently suppressed both HCT116 and HeLa cancer cell growth, and the LD_50_ value was 63.8 and 63.3 μM, respectively. Since the LD_50_ value of compound **1** for HCT116 cells was almost the same as that for HeLa cells, compound **1** might suppress all human cancer cell growth. The cell growth inhibition dose was approximately 1.3 to 5.1-fold higher than the enzyme inhibitory concentration, suggesting that the cause of the cancer cell influence might be the activity of pols. On the other hand, compound **1** did not influence the cell proliferation and growth of human normal cells such as HUVEC (human umbilical vein endothelial cells) and HDF (human dermal fibroblast) (data not shown); therefore, this compound might be a selective anti-cancer chemotherapy agent.

Y-family pols differ from other families in having low fidelity on undamaged templates and in their ability to replicate through damaged DNA. Members of this family are hence called translesion synthesis (TLS) pols [21]. Depending on the lesion, TLS pols can bypass the damage in an error-free or error-prone fashion, the latter resulting in elevated mutagenesis. Xeroderma pigmentosum variant (XPV) patients, for instance, have mutations in the gene encoding pol η, which is error-free for UV lesions. In XPV patients, alternative error-prone pols, e.g. pol ζ (B-family pol), are thought to be involved in mistakes which result in the cancer predisposition of these patients. Other members in humans are pols ι, κ, and Rev1 [6,21]. In *E. coli*, two TLS pols, pol IV (DINB) and pol V (UMUC), are known [21].

As shown in Figure 5B, compound **1** significantly enhanced HeLa cell UV-sensitivity, and the inhibiting clonogenic survival effect of 100 μM of the compound on 10 J/m^2^ UV exposed cells was 4.3-fold stronger than that on non-exposed cells. The LD_50_ value of compound **1** for UV-treated cell growth had the same potency as the IC_50_ values for mammalian Y-family pols. These results suggested that compound **1** inhibited the activities of family Y pols needed for DNA repair, thereby DNA damage must be augmented by this compound and UV-treatment, and then the cancer cells might undergo apoptotic cell death. It has been reported that pol η-deficient human fibroblast cells generated using siRNA were more than 3-fold sensitive to UV and DNA-targeting anticancer agents [22]. This supports the idea that compound **1** might selectively inhibit the activity of Y-family pols in the cells and that inhibitors of Y-family pols may have potential as anti-cancer drugs for clinical radiation therapy or cancer chemotherapy.

## 3. Conclusions

We obtained 3-*O*-methylfunicone (**1**) from a marine fungal strain isolated from Australian sea salt and showed it was a potent inhibitor specific to both mammalian Y-family pols and human cancer cell growth. Compound **1** has extremely high specificity for mammalian pol families, and significantly suppressed UV-treated human cancer cell growth and clonogenic survival. This compound could be a useful molecular tool as a Y-family pol-specific inhibitor in studies to determine the precise roles of the pol family *in vitro*. The products isolated from marine fungal strains, such as compound **1**, may provide valuable information for developing a drug design strategy for anti-cancer chemotherapy agents.

## 4. Experimental

### 4.1. Isolation and Cultivation of Marine Fungal Strains from Australian Sea Salt

**Figure 6.**
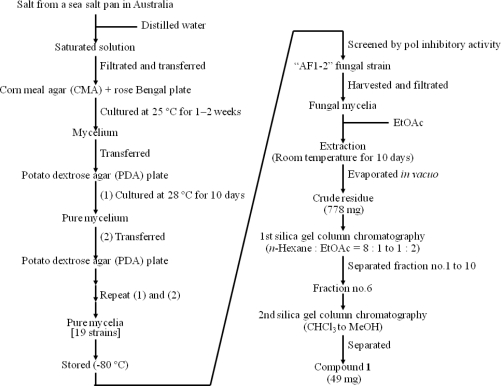
(Left) Isolation methods of marine fungal strains from Australian sea salt. (Right) Purification procedure of a compound with pol inhibitory activity from mycelia of “AF1-2” fungal strain.

Natural salt (75 g) from an Australian sea salt pan was dissolved in distilled water (300 mL), and the saturated solution was filtered through a membrane filter (ADVANTEC, 0.2 μm, 47 mm). The filtered solution was placed on a corn meal agar (CMA) plate (Sigma-Aldrich K. K., Tokyo, Japan) containing rose Bengal (Junnsei Chemical Co., Ltd., Tokyo, Japan), and this plate was cultured for 1–2 weeks at 25 °C. Each grown mycelium on the plate was transferred to individual potato dextrose agar (PDA) (Difco Lab., KS, USA) plates, and cultured at 28 °C for 10 days. Each cultured fungus was transferred to a new PDA plate, and cultured at 28 °C for 10 days. Transformation and culture on PDA plates was repeated two to five times to obtain pure mycelium strains. The number of isolated and purified fungal strains was 19, which were stored at −80 °C (Figure 6, left).

### 4.2. Extraction and Purification of Compound from Marine Fungal Strains

**Figure 7.**
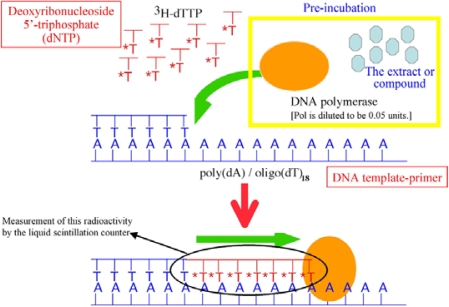
Assay method of pol inhibitor.

**Figure 8.**
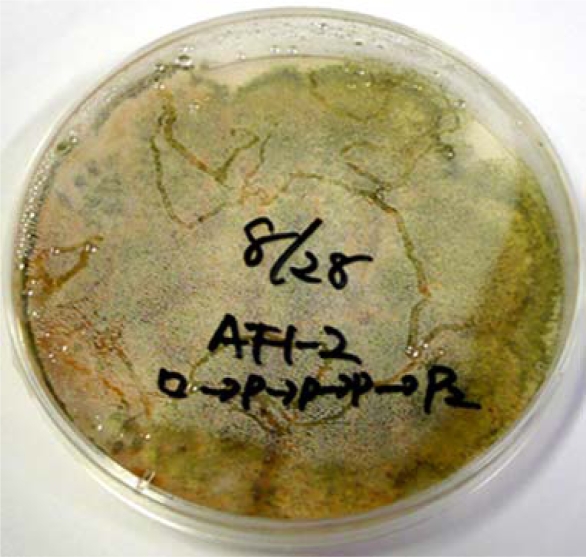
Marine fungal strain AF1-2.

The 19 isolated marine fungal strains were cultured in liquid potato dextrose (PD) medium, and each mycelium was extracted with chloroform/methanol (v/v 1:1). After evaporation of the solvent, the extracts were screened for inhibitory activity against mammalian pols. Pol catalyzes the addition of deoxyribonucleotides to the 3′-hydroxyl terminus of primed double-stranded DNA molecules and the assay method of pol inhibitors is shown in Figure 7. The mycelium extract of strain “AF1-2” displayed the greatest ability to inhibit DNA polymerase activity. An image of the AF1-2 fungal strain is shown in Figure 8. This strain was precultured by transferring a small agar piece to a 3L Erlenmeyer flask containing PD (2.4 g) in H_2_O (100 mL), and then fermented under static conditions in the dark for 3 days. The production culture was initiated by transferring the preculture to production medium [PD (2.4 g) and Daigo’s artificial seawater (36 g; Wako Chemical. Co. Ltd., Tokyo, Japan) in H_2_O (1 L)], and fermentation was carried out at room temperature under static conditions in the dark for 41 days. As shown in Figure 6 (right), the harvested fungal mycelia of AF1-2 were filtered from the cultured broth through cheesecloth, followed by homogenization in EtOAc, and extraction under static conditions at room temperature for 10 days. The extract was evaporated *in vacuo* to obtain crude residue (778 mg), and was separated by silica gel column chromatography (*n*-hexane/EtOAc = 8/1 to 1/2) to give fractions 1–10. Active fraction no. 6 was purified by a 2nd silica gel column chromatography (100% CHCl_3_ to 100% MeOH) to afford the active compound **1** (49 mg).

### 4.3. Structure Determination of Isolated Compound

^1^H- and ^13^C-NMR were recorded on a Bruker DRX400 instrument. Chemical shifts are reported in *δ*, parts per million (ppm), relative to TMS as an internal standard. Mass spectra were obtained on an API QSTAR Pulsar i spectrometer. The molecular formula of compound **1** was determined to be C_20_H_20_O_8_ by the high resolution mass spectrum. From the ^1^H- and ^13^C-NMR spectral data, compound **1** was identified as 3-*O*-methylfunicone (Figure 1). These spectroscopic data were consistent with those previously reported [23]. The analyzed data of compound **1** are shown below: *3-O-Methylfunicone* (**1**). Colorless amorphous solid; ^1^H-NMR (CDCl_3_) *δ*: 8.49 (1H, d, *J* = 0.4 Hz), 7.09 (1H, d, *J* = 2.4 Hz), 6.64 (1H, d, *J* = 2.4 Hz), 6.61 (1H, dq, *J* =16.0, 6.4 Hz), 6.54 (1H, ddd, *J* = 16.0, 1.2, 0.4 Hz), 3.86 (3H, s), 3.81 (3H, s), 3.78 (3H s), 3.73 (3H, s), 1.97 (3H, dd, *J* = 6.4, 1.2 Hz); 13C-NMR (CDCl_3_) *δ*_C_: 191.8, 172.4, 166.4, 160.7, 159.1, 157.3, 154.6, 144.1, 134.9, 129.7, 126.3, 126.1, 118.4, 105.2, 102.9, 60.5, 56.0, 55.5, 52.3, 18.9; HR ESIMS m/z 411.1058 [M+Na]^+^ (calcd for 411.1055).

### 4.4. Materials

Natural salt from a sea salt pan in Australia was supplied by the Research Institute of Salt and Sea Water Science (Kanagawa, Japan). Nucleotides, such as [^3^H]-deoxythymidine 5′-triphosphate (dTTP) (43 Ci/mmol), and chemically synthesized DNA template, such as poly(dA), were purchased from GE Healthcare Bio-Sciences (Little Chalfont, UK). DNA primer, such as oligo(dT)_18_, was customized by Sigma-Aldrich K. K. (Hokkaido, Japan). All other reagents were of analytical grade and were purchased from Wako Chemical Industries (Osaka, Japan).

### 4.5. Enzymes

Pol α was purified from calf thymus by immuno-affinity column chromatography, as described by Tamai *et al*. [24]. Recombinant rat pol β was purified from *E. coli* JMpβ5, as described by Date *et al*. [25]. The human pol γ catalytic gene was cloned into pFastBac. Histidine-tagged enzyme was expressed using the BAC-TO-BAC HT Baculovirus Expression System according to the supplier’s manual (Life Technologies, MD, USA) and purified using ProBoundresin (Invitrogen Japan, Tokyo, Japan) [26]. Human pols δ and ɛ were purified by nuclear fractionation of human peripheral blood cancer cells (Molt-4) using the second subunit of pols δ and ɛ-conjugated affinity column chromatography, respectively [27]. A truncated form of human pol η (residues 1–511) tagged with His_6_ at its C-terminal was expressed in *E. coli* cells and purified as described by Kusumoto *et al*. [28]. A recombinant mouse pol ι tagged with His_6_ at its C-terminal was expressed and purified by Ni-NTA column chromatography as described elsewhere (Masutani *et al*., in preparation). A truncated form of pol κ (residues 1–560) with 6 x His-tags attached at the C-terminus was overproduced in *E. coli* and purified as described by Ohashi *et al*. [29]. Recombinant human His-pol λ was overexpressed and purified according to a method described by Shimazaki *et al*. [30]. Fish pol δ was purified from the testis of cherry salmon (*Oncorhynchus masou*) [31]. Fruit fly pols α, δ and ɛ were purified from early embryos of *Drosophila melanogaster*, as described by Aoyagi *et al*. [32,33]. Pol α from a higher plant, cauliflower inflorescence, was purified according to the methods outlined by Sakaguchi *et al*. [34]. Recombinant rice (*Oryza sativa* L. cv. Nipponbare) His-pol λ was overexpressed and purified according to a method described by Uchiyama *et al*. [35]. Calf thymus TdT and bovine pancreas DNase I were obtained from Stratagene Cloning Systems (La Jolla, CA, USA). HIV-1 reverse transcriptase (recombinant) and the Klenow fragment of pol I from *E. coli* were purchased from Worthington Biochemical Corp. (Freehold, NJ, USA). T4 pol, *Taq* pol, T7 RNA polymerase and T4 polynucleotide kinase were purchased from Takara Bio (Tokyo, Japan). Purified human placenta DNA topoisomerases I and II were purchased from TopoGen, Inc. (Columbus, OH, USA).

### 4.6. DNA Polymerase Assays

The reaction mixtures for pol α, pol β, plant pols and prokaryotic pols were described previously [7,8]; those for pol γ, and pols δ and ɛ were as described by Umeda *et al*. [26] and Ogawa *et al*. [36], respectively. The reaction mixtures for pols η, ι and κ were the same as for pol α, and the reaction mixture for pol λ was the same as for pol β. For pols, poly(dA)/oligo(dT)_18_ (A/T = 2/1) and dTTP were used as the DNA template-primer and nucleotide (*i.e.*, dNTP) substrate, respectively. For HIV-1 reverse transcriptase, poly(rA)/oligo(dT)_18_ (A/T = 2/1) and dTTP were used as the DNA template-primer and nucleotide substrate, respectively. For TdT, oligo(dT)_18_ (3′-OH) and dTTP were used as the DNA primer and nucleotide substrate, respectively.

The compounds were dissolved in distilled dimethyl sulfoxide (DMSO) at various concentrations and sonicated for 30 sec. Aliquots of 4 μl sonicated samples were mixed with 16 μl of each enzyme (final amount 0.05 units) in 50 mM Tris-HCl (pH7.5) containing 1 mM dithiothreitol, 50% glycerol and 0.1 mM EDTA, and kept at 0 °C for 10 min. These inhibitor-enzyme mixtures (8 μl) were added to 16 μl of each of the enzyme standard reaction mixtures (final concentration of 50 mM Tris-HCl [pH 7.5], 1 mM dithiothreitol, 1 mM MgCl_2_, 15% glycerol, 10 μM poly(dA)/oligo(dT)_18_ and 10 μM [^3^H]-dTTP), and incubation was carried out at 37 °C for 60 min, except for *Taq* pol, which was incubated at 74 °C for 60 min. Activity without the inhibitor was considered 100%, and the remaining activity at each concentration of the inhibitor was determined relative to this value. One unit of pol activity was defined as the amount of enzyme that catalyzed the incorporation of 1 nmol dNTP (*i.e.*, dTTP) into synthetic DNA template-primers in 60 min at 37 °C under the normal reaction conditions for each enzyme [7,8].

### 4.7. Other DNA Metabolic Enzymes Assays

The activities of calf primase of pol α, human telomerase, T7 RNA polymerase, mouse IMP dehydrogenase, human DNA topoisomerases I and II, T4 polynucleotide kinase and bovine DNase I were measured in standard assays according to the manufacturer’s specifications, as described by Tamiya-Koizumi *et al*. [37], Oda *et al*. [38], Nakayama *et al*. [39], Mizushina *et al*. [40], Ishimaru *et al*. [41], Soltis *et al*. [42] and Lu *et al*. [43], respectively.

### 4.8. Investigation of Growth on Cultured Human Cancer Cells

Human colon carcinoma cell line, HCT116, was a kind gift from Dr. Bert Vogelstein (Johns Hopkins University, Baltimore, MO, USA). Human cervix carcinoma cell line, HeLa, was obtained from Health Science Research Resources Bank (Osaka, Japan). The cells were maintained in McCoy’s 5A medium supplemented with 10% FBS, sodium bicarbonate (2 g/L) and streptomycin (100 μg/mL). The cells were cultured at 37 °C in standard medium in a humidified atmosphere of 5% CO_2_-95% air. The cytotoxicity of the compound was investigated as follows: high concentrations (10 mM) of the compounds were dissolved in DMSO and stored. Approximately 5 × 10^3^ cells per well were inoculated in 96-well micro plates, then the compound stock solution was diluted to various concentrations, and applied to each well. After incubation for 24 h, the survival rate was determined by MTT (3-(4,5-dimethylthiazol-2-yl)-2,5-diphenyl tetrazolium bromide) assay [44].

### 4.9. UV-Treated Clonogenic Assay

For clonogenic assay, 600 cells of HeLa were seeded on 10-cm plates and allowed to grow for 24 h before 10 J/m^2^ UV irradiation treatment. The cells were then treated with various concentrations of compound **1** for 24 h. The cells were then washed with PBS and fresh medium was added for an additional 7-day incubation in a humidified 10% CO_2_ incubator at 37 °C. After 7 days, the cultured cells were stained with 0.5% methylene blue in 50% ethanol to visualize the colonies. The number of colonies in each treatment group was counted, and the cell survival data were presented as percentage of the colony numbers of treated/untreated cells.
